# Hidradenitis suppurativa in AIDS

**DOI:** 10.4103/2589-0557.69002

**Published:** 2010

**Authors:** Ravi Khambhati, Priyanka Singhal, Y. S. Marfatia

**Affiliations:** Department of Skin VD, Government Medical College and SSG Hospital, Vadodara, India

**Keywords:** AIDS, Hidradenitis suppurativa, Apocrine gland

## Abstract

Hidradenitis suppurativa (HS) is a disorder of the terminal follicular epithelium in the apocrine gland-bearing skin, characterized by comedo-like follicular occlusion, chronic relapsing inflammation, mucopurulent discharge, and progressive scarring. In this study, we report a case of 35-year-old HIV-positive man with recurrent nodular skin lesions with foul smelling discharge over face, gluteal region, thighs, and axilla. This case is unique because of its association with HIV leading to atypical manifestations and therapeutic challenges.

## INTRODUCTION

Hidradenitis suppurativa (HS) is a disorder of the terminal follicular epithelium in the apocrine gland-bearing skin. It is characterized by comedo-like follicular occlusion, chronic relapsing inflammation, mucopurulent discharge, and progressive scarring.[1] HIV-induced immunological changes significantly alter the clinical course of chronic skin conditions. Little is known about the association of HIV and HS.

## CASE REPORT

**Figure 1:**
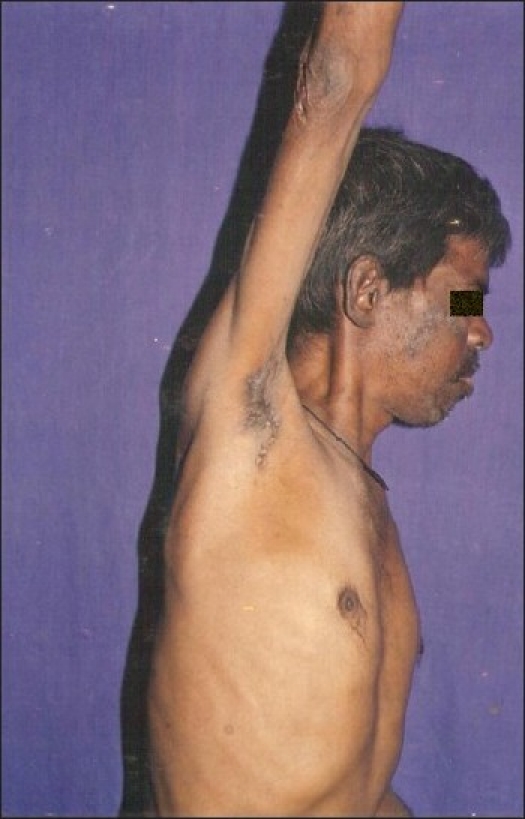
Nodules over axilla

**Figure 2:**
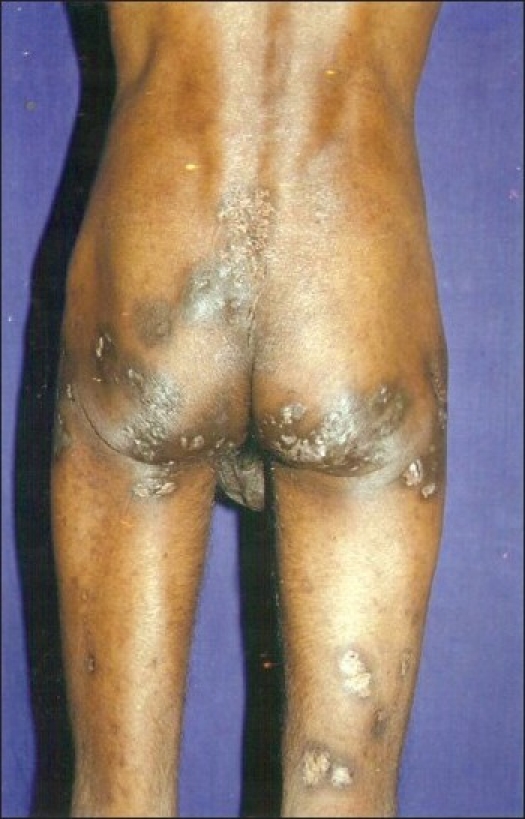
Nodules and scarring over gluteal region and thighs

**Figure 3:**
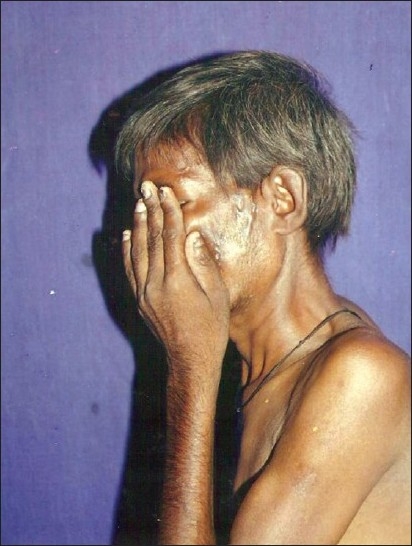
Nodules over face

A 35-year-old man is presented with skin lesions over face, thighs, gluteal region, and axilla [Figures 1–3]. As per the patient, he developed nodular skin lesions with foul smelling discharge over face, gluteal region, thighs, and axilla 2 years back. The lesions progressed to form ulcer and scars. The condition responded partially to antibiotic treatment, and the patient suffered from recurrent lesions. He also complained of diarrhea, prolonged fever, and weight loss >10%. Patient had a history of multiple unsafe sexual exposure. There was no history of genital ulcer or discharge.

General examination revealed marked wasting. Inguinal lymphnodes were enlarged, discrete, and nontender. Systemic examination was normal. Multiple nodules, comedons, discharging sinuses, and scarring were present over face, axilla, gluteal region, and posterior thighs. *Laboratory investigations*: complete blood picture revealed HB 10 g%, total count was low 3000/cumm, differential count showed predominant neutrophils 86/13/1/0, and patient had raised ESR: 70 mm/h. *Staphylococcus aureus* was isolated in culture. ELISA for HIV was reactive. Due to resource constraints, CD4 count could not be carried out. X-ray and USG abdomen were carried out to study tuberculosis were normal. FNAC of inguinal lymphnodes showed reactive lymphadenitis.

Differential diagnosis considered were HS, *Staphylococcal folliculitis, and Eosinophilic folliculitis*.

A confirmatory biopsy results showed folliculitis and perifolliculitis with dilated infundibulum containing keratinous material and inflammatory dermis and heavy, mixed inflammatory cell infiltrate consisting of neutrophils, lymphocyte in lower half of dermis suggestive of HS.

Patient was put on oral doxycycline 100 mg BID and ibuprofen. Dapsone 100 mg was added. However, there was partial response with recurrence. Patient had AIDS defining illness, but ART could not be offered as he presented to us in Pre-ART era.

## DISCUSSION

Hidradenitis suppurativa (acne inversa, Pyoderma fistulans significa, and Verneuil’s disease) is a chronic suppurating infection that affects the apocrine glands of the axilla, groin, and the perineum. The disease begins with the obstruction of the apocrine gland duct, resulting in the infection of the retained secretions.[2] Following gland obstruction, there is rupture of the gland with spread of infection into the dermis leading to abscess formation and involvement of other apocrine glands. There is the formation of multiple intradermal abscesses, which lead to the development of multiple sinuses, fistulae, and scarring of the skin. Occasionally, the disease extends beyond the dermis into the subcutaneous fat, fascia, and muscle. The lesions present in the form of nodules, discharging sinuses, and comedones predominantly over axillae, anogenital region.[3] Atypical sites were involved in this patient such as face, thighs which could be due to HIV-related immunosuppression. Etiology may also have an endocrinal component, and HIV-associated endocinopathies may alter the course of the disease. Association of AIDS with a chronic skin condition like HS is a therapeutic challenge. Oral retinoids (isotretinoin) have a proven value in unresponsive cases. However, because of financial constraints it could not be used in our patient. Use of isotretinoin for HS in HIV-positive patients on ART needs regular monitoring due to adverse effects such as raised triglycerides and effect on liver transaminases.[4] Other therapeutic options include use of antibiotics such as flucloxacillin or prolonged courses of tetracycline or metronidazole (minimum 3 months) for their anti-inflammatory action. Combination of clindamycin and rifampicin may be effective. Surgical management like incision and drainage of abscesses and excision of scars need to be individualized.
